# Curcumin in combination with homoharringtonine suppresses lymphoma cell growth by inhibiting the TGF-β/Smad3 signaling pathway

**DOI:** 10.18632/aging.203319

**Published:** 2021-07-29

**Authors:** Yu Zhang, Jingjing Xiang, Ni Zhu, Hangping Ge, Xianfu Sheng, Shu Deng, Junfa Chen, Lihong Yu, Yan Zhou, Jianping Shen

**Affiliations:** 1 Department of Hematology, The First Affiliated Hospital of Zhejiang Chinese Medical University, Hangzhou, Zhejiang 310006, PR China; 2 The First Medical College of Zhejiang Chinese Medical University, Hangzhou, Zhejiang 310053, PR China

**Keywords:** lymphomas, homoharringtonine, curcumin, apoptosis, combination treatment

## Abstract

Both homoharringtonine (HHT) and curcumin exhibit anti-proliferative effects on lymphoma cells, but the effects of combined HHT and curcumin treatment remain unclear. Here, we investigated the effects of HHT/curcumin combination on the proliferation, apoptosis, and invasion in lymphoma cells. CCK-8, flow cytometry, and transwell assays were used to assess proliferation, apoptosis, and invasion of U937 and Raji cells. p-Smad3, E-cadherin, and N-cadherin expression were also measured in Raji cells using Western blot assays. Combination of HHT and curcumin synergistically inhibited U937 and Raji cell proliferation and invasion. In addition, the combination treatment markedly increased apoptosis of Raji cells as evidenced by increased Bax, cleaved caspase 3, and cleaved caspase 9 expression. Meanwhile, the combination treatment promoted anti-tumor mechanisms in Raji cells as indicated by decreases in p-Smad3 and N-cadherin and increases in E-cadherin. *In vivo* experiments showed that the combination treatment suppressed tumor growth in a mouse Raji xenograft model. Our findings indicate that combination of HHT and curcumin inhibited lymphoma cell growth by downregulating the TGF-β/Smad3 pathway. These results suggest that HHT combined with curcumin might be a promising therapeutic approach for the treatment of lymphoma.

## INTRODUCTION

Lymphomas are a heterogeneous group of lymphoid malignancies [1]. Mature lymphoid neoplasms are classified by the WHO as either non-Hodgkin’s lymphoma (NHL) or Hodgkin’s lymphoma (HL) [1, 2]. Burkitt’s lymphoma is a highly invasive NHL that primarily affects children and adolescents [3, 4]. Burkitt’s lymphoma is also an aggressive B cell acute lymphoblastic leukemia [5]. At present, chemotherapy, immunotherapy, and radiotherapy are used to treat lymphomas [6–8]. However, the prognosis of lymphoma patients remains poor due to chemoradiotherapy resistance [9, 10]. Therefore, the development of novel treatment strategies has become a major focus of research.

Homoharringtonine (HHT) is a compound that was initially extracted from the *Cephalotaxus hainanensis* Li plant used in traditional Chinese medicine [11]. HHT was approved by the US Food and Drug Administration (FDA) for treatment of patients with chronic myeloid leukemia in 2012 [12]. Since the 1970s, HHT has been used in the treatment of hematological malignancies in China [13, 14]. Nguyen et al. found that HHT combined with bortezomib could kill diffuse large B-cell lymphoma (DLBCL) cells [15]. In addition, Klanova et al. found that HHT significantly inhibited cell growth in murine xenograft models of DLBCL [16].

Curcumin is a polyphenol derived from the rhizomes of the Chinese medicinal herb *Curcuma Longa* L [17, 18]. Curcumin has strong anti-oxidative and anti-inflammatory activities [19], and studies have shown that curcumin exhibits strong anti-tumor effects on colorectal, prostate, breast, and gastric cancers [20–23]. Guorgui et al. found that curcumin exhibited an anti-proliferative effect in HL [24]. In addition, curcumin could be taken up by cells in a 1/20 ratio and then induced the apoptosis of cancer cells [25–28]. Although HHT and curcumin have shown anti-tumor effects in lymphoma cells, little is known about the effects of a combination of these two compounds.

Epithelial-mesenchymal transition (EMT) is an essential process for acquisition of aggressiveness and metastatic capacity in tumor, which is characterized by loss of epithelial markers (such as E-cadherin) and gain of mesenchymal markers (such as N-cadherin). [29]. In addition, transforming growth factor-beta1 (TGF-β1)/Smads signaling has been found to exert an important role in EMT [30]. Evidences have found that activation of TGF-β/Smad3 signaling pathway could promote the metastasis and EMT in tumor cells [31, 32]. In this study, we found that HHT combined with curcumin could exert tumoricidal effects in lymphoma cells *in vitro* and *in vivo* via inhibition of TGF-β/Smad3 signaling pathway. These results suggested that HHT combined with curcumin might be a promising therapeutic approach for the treatment of lymphoma.

## RESULTS

### Combination of curcumin and HHT synergistically inhibited lymphoma cell proliferation

**Figure 1:**
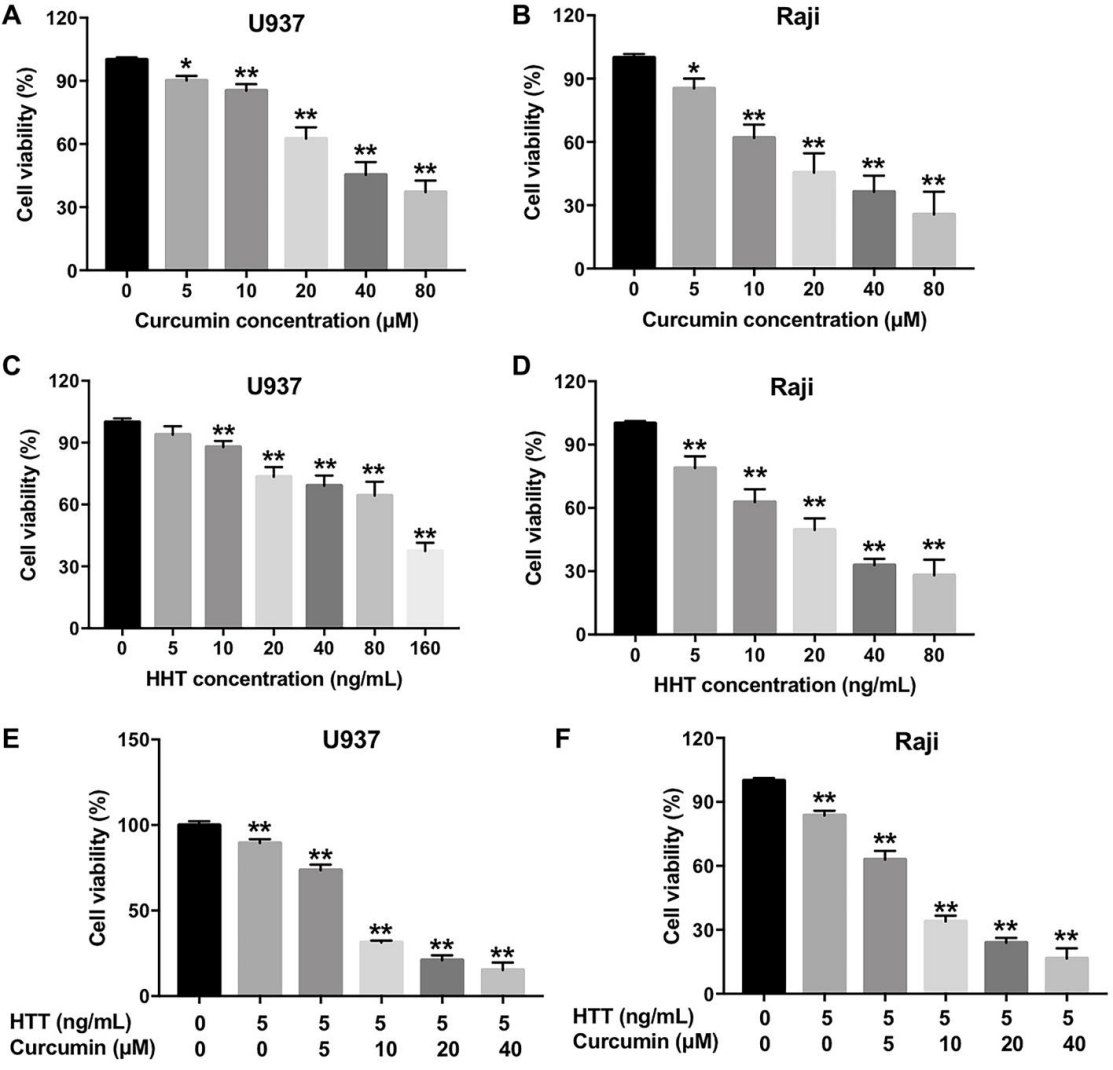
**Combination of curcumin with HHT synergistically inhibited lymphoma cell proliferation.** (**A**) U937 or (**B**) Raji cells were treated with 0, 5, 10, 20, 40, or 80 μM curcumin for 72 h. CCK-8 assay was used to measure cell viability. (**C**) U937 or (**D**) Raji cells were treated with 0, 5, 10, 20, 40, 80, or 160 ng/mL HHT for 72 h. CCK-8 assay was used to measure cell viability. (**E**) U937 or (**F**) Raji cells were treated with 5 ng/mL HHT plus curcumin (0, 5, 10, 20, or 40 μM) for 72 h. CCK-8 assay was used to measure cell viability. ^*^P < 0.05, ^**^P < 0.01 compared to control group.

A CCK-8 assay was used to determine the effects of curcumin and HHT on the viability of U937 and Raji cells. As shown in Figure 1A–1D, curcumin or HHT treatment inhibited U937 and Raji cell viability in a dose-dependent manner. In addition, combined HHT and curcumin treatment significantly inhibited U937 and Raji cell viability (Figure 1E and 1F). The IC_50_ values of curcumin when administered alone were 39.52 μM and 19.79 μM in U937 and Raji cells, respectively; when HHT (5 ng/mL) was combined with curcumin, the latter’s IC_50_ value was decreased to 7.14 μM and 6.71 μM, respectively (Table 1). Moreover, the CI values for the combination of HHT and curcumin in U937 cells were less than 0.4, which indicated a strong synergistic effect (Table 1). The CI values for combined HHT and curcumin in Raji cells were less than 0.6, also indicating a synergistic effect (Table 1). These data indicate that combination of curcumin and HHT synergistically inhibited lymphoma cell proliferation.

**Table 1:** Evaluation of combination of HHT with curumin in U937 and raji cells (72 h treatment).

**Drug combination**	**U937 cells**		**Raji cells**	
	**IC 50 value**	**CI values**	**IC 50 value**	**CI values**
Curumin (range 0 from 40 μM)	IC50 = 39.52 μM	–	IC50 = 19.79 μM	–
Curumin + 5 ng/mL HHT	IC50 = 7.14 μM	0.23	IC50 = 6.71 μM	0.59

### Combination of curcumin and HHT induced apoptosis and suppressed migratory and invasive ability in lymphoma cells

**Figure 2:**
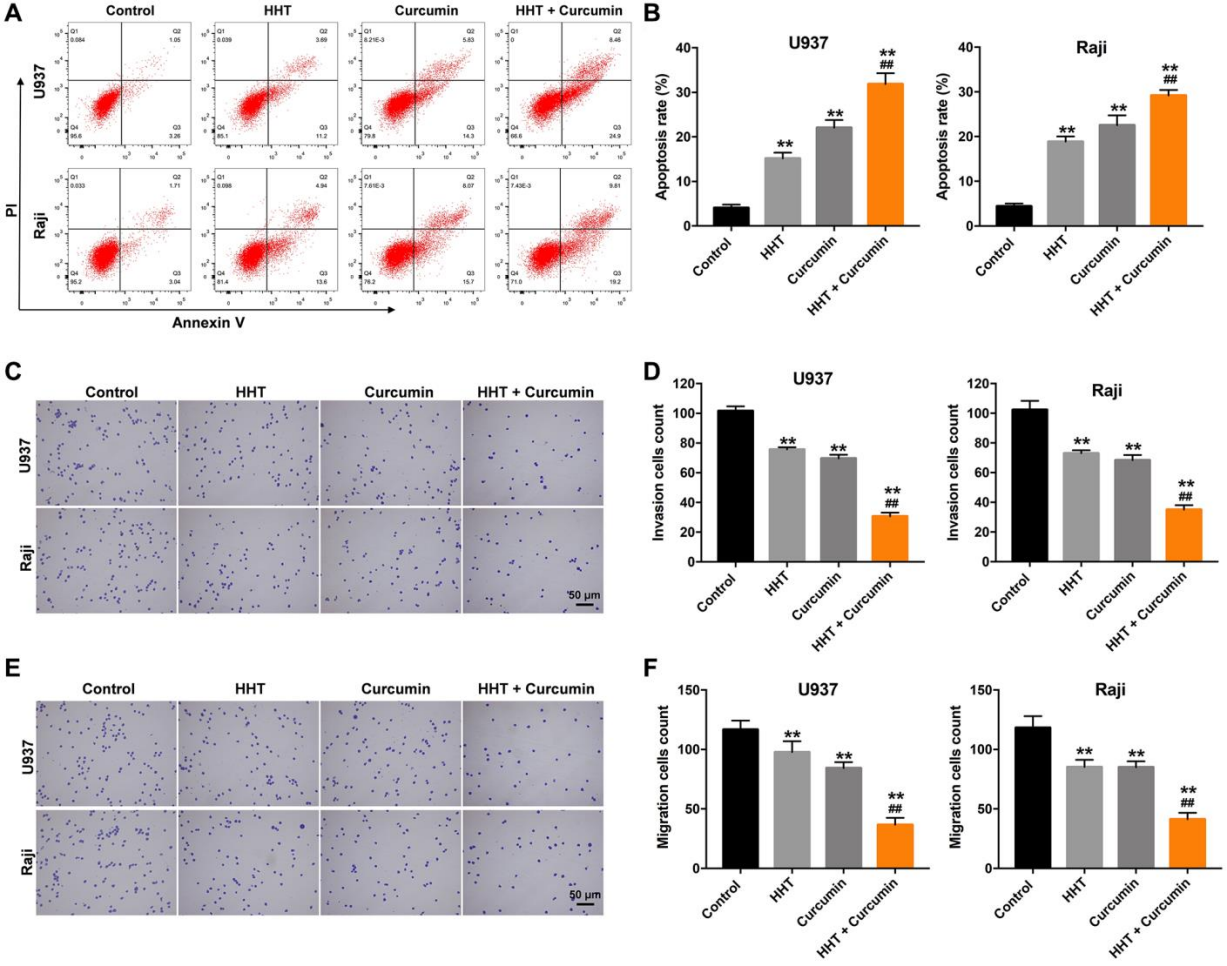
**Combination of curcumin with HHT induced apoptosis and suppressed invasive ability of lymphoma cells.** (**A**, **B**) U937 and Raji cells were treated with 5 ng/mL HHT or/and 10 μM curcumin for 72 h. Apoptotic cells were quantified by flow cytometry. (**C**, **D**) U937 and Raji cells were treated with 5 ng/mL HHT or/and 10 μM curcumin for 24 h. Cell invasion was assessed in a transwell invasion assay. (**E**, **F**) Cell migration was assessed in a transwell migration assay. ^**^P < 0.01 compared to control group; ^##^*P* < 0.01 compared to HHT group.

A flow cytometry assay was used to investigate the effects of curcumin and HHT on apoptosis in lymphoma cells. As shown in Figure 2A and 2B, treatment with either HHT (15.1%) or curcumin (22.0%) significantly increased the apoptosis of U937 cells compared with control (4.1%) group, and HHT (18.9%) or curcumin (22.5%) notably increased the apoptosis of Raji cells compared with control (4.4%) group. As expected, the apoptotic cells increased up to 31.8% for U937 cell treated with the combination (5 ng/mL HHT plus 10 μM curcumin), compared to 15.1% with HHT (5 ng/mL) alone, and the apoptotic cells increased up to 29.2% for Raji cell treated with the combination (5 ng/mL HHT plus 10 μM curcumin), compared to 18.9% with HHT (5 ng/mL) alone (Figure 2A and 2B). In addition, HHT treatment showed 25.0% and 28.6% decreases in invasion cells count in U937 and Raji cells, respectively, and curcumin caused 31.3% and 33.2% decreases, while combined HHT and curcumin treatment produced 69.8% and 65.8% decreases in invasion cells count after 24 h of treatment (Figure 2C and 2D). Meanwhile, HHT treatment showed 16.3% and 28.2% decreases in migration cells count in U937 and Raji cells, respectively, and curcumin caused 27.7% and 28.2% decreases, while the combined treatment produced 68.6% and 65.1% decreases in migration cells count (Figure 2E and 2F). These results indicate that the combination of curcumin and HHT induced apoptosis and suppressed migratory and invasive ability in lymphoma cells.

### Combination of curcumin with HHT induced apoptosis in lymphoma cells via the mitochondrial-mediated apoptosis pathway

**Figure 3:**
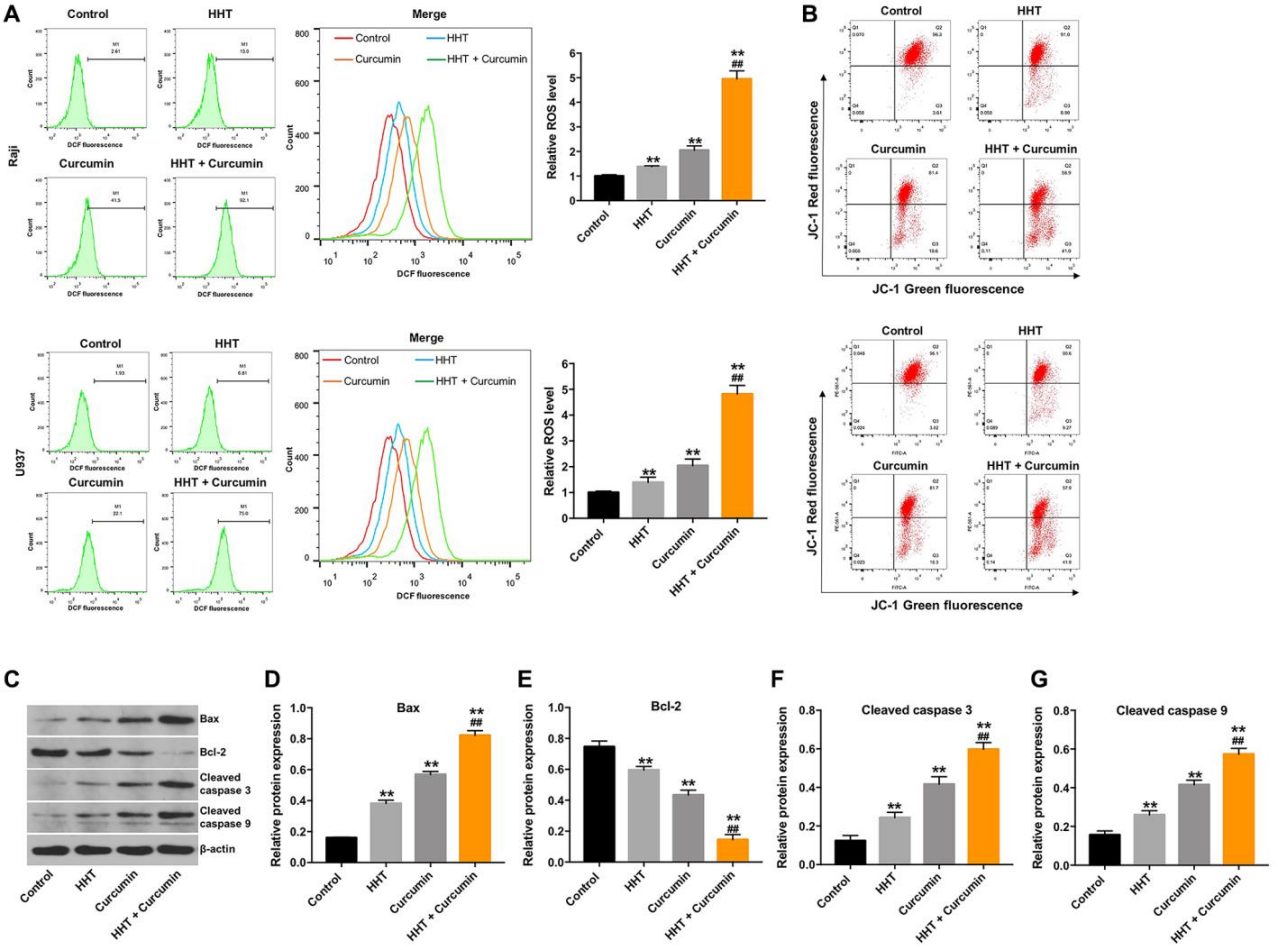
**Combination of curcumin with HHT induced apoptosis of lymphoma cells via the mitochondrial-mediated apoptosis pathway.** Raji and U937 cells were treated with 5 ng/mL HHT or/and 10 μM curcumin for 72 h. (**A**) Intracellular ROS generation was measured by DCF fluorescence. (**B**) MMP loss was determined via JC-1 staining. (**C**) Bax, Bcl-2, cleaved caspase 3, and cleaved caspase 9 expression were measured in Raji cells using Western blotting. (**D**, **E**, **F**, **G**) Relative cellular Bax, Bcl-2, cleaved caspase 3, and cleaved caspase 9 expression normalized to β-actin. ^**^P < 0.01 compared to control group; ^##^*P* < 0.01 compared to HHT group.

To assess whether lymphoma cell death induced by HHT/curcumin combination was accompanied by enhanced ROS generation, Raji cells were stained with DCFH-DA. As shown in Figure 3A, combination of HHT and curcumin remarkedly increased ROS generation in Raji cells (4.95 fold) and U937 cells (4.82 fold) compared to the HHT (1.38 fold and 1.39 fold, respectively) alone treatment group (Figure 3A). In addition, JC-1 staining indicated that the combination treatment obviously decreased MMP in Raji and U937 cells (Figure 3B). Meanwhile, combination of HHT and curcumin markedly increased Bax, cleaved caspase 3, and cleaved caspase 9 expression and decreased Bcl-2 expression in Raji cells (Figure 3C–3G). These data demonstrate that combination of curcumin and HHT induced apoptosis of lymphoma cells via the mitochondrial-mediated apoptosis pathway.

### Combination of curcumin and HHT inhibited the EMT in lymphoma cells by inhibiting the TGF-β/Smad3 signaling pathway

**Figure 4:**
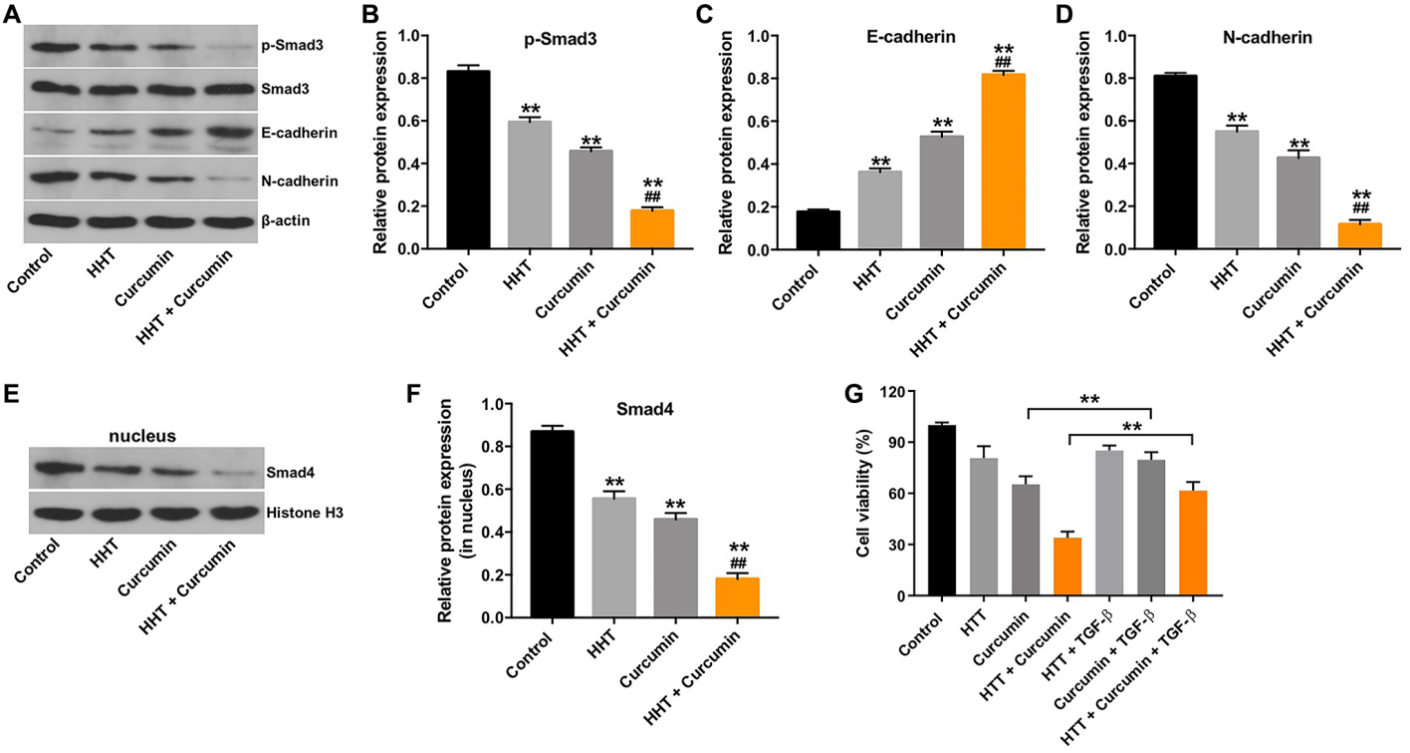
**Combination of curcumin with HHT inhibited the EMT in lymphoma cells by inhibiting the TGF-β1/Smad3 signaling pathway.** Raji cells were treated with 5 ng/mL HHT or/and 10 μM curcumin for 72 h. (**A**) p-Smad3, Smad3, E-cadherin, and N-cadherin expression were measured in Raji cells using Western blotting. (**B**, **C**, **D**) Relative cellular p-Smad3, E-cadherin, and N-cadherin normalized to Smad3, β-actin, and β-actin, respectively. (**E**, **F**) Nuclear Smad4 expression in Raji cells was measured by Western blotting. Relative Smad4 expression was determined by normalizing to Histone H3. ^**^P < 0.01 compared to control group; ^##^*P* < 0.01 compared to HHT group. (**G**) Raji cells were treated with HHT and curcumin for 72 h or treated with HHT, curcumin and TGF-β. CCK-8 assay was used to measure cell viability. ^**^P < 0.01.

TGF-β/Smad signaling participates in various biological processes, including cancer cell growth, migration, and invasion [33]. We therefore assessed p-Smad3, E-cadherin, and N-cadherin expression in Raji cells using a Western blotting assay. As indicated in Figure 4A–4D, combination of HHT and curcumin significantly decreased p-Smad3 and N-cadherin levels and markedly increased E-cadherin levels in Raji cells. Meanwhile, the combination treatment decreased nuclear accumulation of Smad4 in Raji cells (Figure 4E and 4F). To investigate whether TGF-β signaling is involved in combination treatment-mediated lymphoma cell growth, rescue experiments were performed. As shown in Figure 4G, the inhibitory effect of combination treatment on the viability of Raji cells was reversed by the administration of TGF-β. These data suggest that combination of curcumin and HHT inhibited lymphoma cell growth by inhibiting the TGF-β/Smad3 signaling pathway.

### Combination of curcumin and HHT inhibited tumorigenesis in Raji xenograft *in vivo*

**Figure 5:**
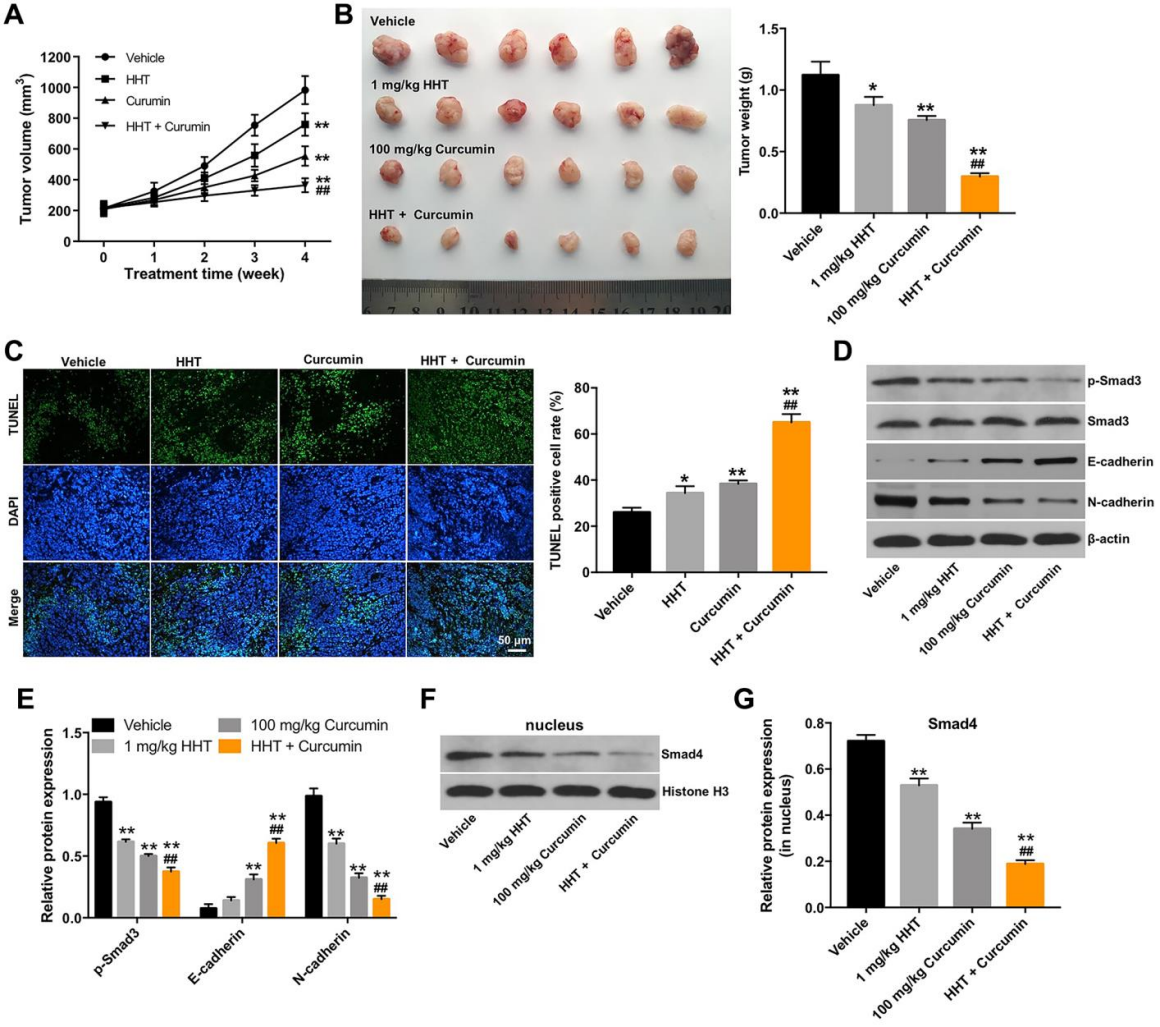
**Combination of curcumin with HHT inhibits tumorigenesis in Raji xenografts *in vivo*.** (**A**) Tumor volume was calculated. (**B**) Raji xenograft tumors were excised and photographed. Tumor weights were calculated for each group of mice. (**C**) A TUNEL assay was used to assess cell apoptosis in tumor tissues. (**D**) p-Smad3, Smad3, E-cadherin and N-cadherin expressions were measured in tumor tissues using Western blotting. (**E**) Relative p-Smad3, E-cadherin and N-cadherin expressions in tumor tissues normalized to Smad3, β-actin and β-actin. (**F**, **G**) Smad4 expression in tumor tissues was measured by Western blotting. Relative Smad4 was determined by normalizing to Histone H3. ^*^P < 0.05, ^**^P < 0.01 compared to control group; ^##^*P* < 0.01 compared to HHT group.

Next, we further assessed the effects of HHT combined with curcumin in a mouse Raji xenograft *in vivo* model. As shown in Figure 5A and 5B, the combination treatment markedly decreased the tumor volume and tumor weight of transplanted Raji tumors *in vivo*. In addition, a TUNEL assay revealed that combined treatment notably increased cell apoptosis in tumor tissues compared to the HHT alone treatment group (Figure 5C). Meanwhile, Western blots indicated that the combination treatment decreased the expressions of p-Smad3 and N-cadherin, increased the expression of E-cadherin, and reduced nuclear accumulation of Smad4 in tumor tissues (Figure 5D–5G). These results indicate that combination of curcumin with HHT inhibited tumorigenesis in Raji xenografts *in vivo*.

## DISCUSSION

In this study, we found that combination of curcumin with HHT synergistically inhibited lymphoma cell proliferation. Moreover, this combination treatment suppressed migration and invasion and induced apoptosis of lymphoma cells by inhibiting the TGF-β1/Smad3 pathway.

HHT is widely used as an antineoplastic drug in the treatment of hematological malignancies [34]. In addition, curcumin inhibits lymphoma cell growth [35]. Previous studies have shown that combining anti-cancer agents can reduce the toxicity and improve the effectiveness of anti-cancer therapies [35, 36]. In particular, addition of curcumin can enhance the efficacy of conventional anticancer therapies [37, 38]. For example, Guo et al. found that curcumin increased the efficacy of doxorubicin treatment in lymphoma cells by inducing apoptosis [35]. In addition, Li et al. found that combination of HHT with triptolide synergistically inhibited KG-1a cell proliferation [39]. In this study, we found that combination of curcumin with HHT synergistically inhibited lymphoma cell proliferation. In addition, the combined treatment with HHT and curcumin induced apoptosis and inhibited invasive ability of lymphoma cells to a significantly greater extent than treatment with either curcumin or HHT alone. These data indicate that combination of curcumin and HHT could suppress lymphoma cell growth to a greater extent than either compound alone.

TGF-β1 is a pleiotrophic cytokine that plays roles in cell differentiation, growth, and apoptosis [40]. Chen et al. found that exogenous TGF-β1 suppressed cellular growth in B-cell lymphoma [41]. However, Jung et al. found that TGF-β1 promoted lymphoma cell survival by regulating Fas-mediated apoptosis signaling [40]. In addition, Chang et al. found that suppression of TGF-β1 induced apoptosis in T cell lymphoma [42]. TGF-β might therefore function as both a pro- and anti-apoptotic regulatory factor in lymphoma cells. Zhang et al. found that curcumin inhibited metastasis in papillary thyroid carcinoma cells by downregulating the TGF-β/Smad3 pathway [43]. In addition, Yin et al. demonstrated that combination of curcumin with oxaliplatin significantly suppressed colorectal cancer cell growth by suppressing TGF-β/Smad3 signaling [44]. Consistent with these previous findings, we found that combination treatment decreased Smad3 phosphorylation and reduce nuclear accumulation of Smad4 in lymphoma cells *in vitro* and *in vivo*. These data suggest that combination of curcumin and HHT may suppress lymphoma cell growth and EMT by downregulating the TGF-β/Smad3 pathway. Zhang et al. found that curcumin could suppress the proliferation of endometrial carcinoma cells by downregulating ERK/c-Jun signaling [45]. In addition, curcumin could inhibit the proliferation and EMT in colon cancer cells via inactivating the Wnt signaling pathway [46]. Thus, further study is needed to identify whether curcumin enhance the anti-tumor effect of HHT on lymphoma cells via mediating ERK/c-Jun signaling or Wnt signaling.

In conclusion, combination treatment of curcumin with HHT exerted anti-lymphoma effects in Raji cells by inactivating the TGF-β/Smad3 pathway. Combined curcumin and HHT treatment might therefore be a promising therapeutic approach for the treatment of lymphomas. However, in the future, further investigation into the mechanistic aspects of the synergism between HHT and curcumin may provide novel therapeutic approaches for the treatment of lymphomas.

## MATERIALS AND METHODS

### Cell culture

The human leukemic monocyte lymphoma cell line U937 and Burkitt's lymphoma cell line Raji were purchased from American Type Culture Collection (ATCC, Rockville, MD, USA). Cells were cultured in Dulbecco's modified Eagle's medium (DMEM, Thermo Fisher Scientific, Waltham, MA, USA) supplemented with 10% fetal bovine serum (FBS, Thermo Fisher Scientific), 100 μg/mL of penicillin, and 100 units/mL of streptomycin. Cells were incubated in a humidified incubator containing 5% CO_2_ at 37°C. Curcumin was purchased from MedChem Expression Co. Ltd (Shanghai, China).

### Cell Counting Kit-8 assay

The Cell Counting Kit-8 (CCK8) (Dojindo Laboratories, Japan) was used to assess cell viability after 3 days of curcumin or HHT treatment. U937 and Raji cells (5 × 10^3^ cells per well) were plated onto 96-well plates at 37°C, and 10 μL of CCK-8 reagent was added to each well followed by incubation for 2 h. Subsequently, a Multiskan™ FC Microplate Photometer (Thermo Fisher Scientific) was used to determine the optical density (OD) of each well at 450 nm.

### Combination studies

The Chou–Talalay method was used to calculate combination index (CI) values for the drug combination [47]. U937 and Raji cells were exposed to solutions containing curcumin (0, 5, 10, 20, or 40 μM) combined with HHT (5 ng/mL). The CI value for the combination of curcumin and HHT in lymphoma was defined as CI = DA/ICx, A + DB/ICx, B [48].

### Flow cytometry analysis

Analysis of apoptosis rates in U937 and Raji cells was carried out using the fluorescein isothiocyanate (FITC) Annexin V Apoptosis Detection Kit (BD Biosciences, Franklin Lake, NJ, USA). Cells (5 × 10^5^/ml) were resuspended in binding buffer and then stained with Annexin V-FITC and propidium iodide (PI) for 20 min in the dark. Numbers of apoptotic cells were then measured using a BD FACSCalibur flow cytometer (BD Biosciences) and analyzed using the CellQuest Pro software (BD Biosciences).

### Transwell assays

Cell migration or invasion was examined using Matrigel-uncoated or Matrigel-coated transwell inserts (8 μm pore size, Corning, USA). U937 and Raji cells (4 × 10^4^ cells/well) were suspended in 150 μL serum-starved culture medium and then added to the upper chambers, and the lower chambers were filled with 700 μL of DMEM medium containing 10% FBS. Twenty-four hours later, cells that had moved through the transwell membrane and invaded the lower chamber were fixed with 70% methanol and stained with 1% crystal violet. The migrated or invaded cells were imaged using a fluorescence microscope and counted in five randomly selected fields.

### ROS detection

The DCFDA/H2DCFDA-Cellular ROS Assay kit (Abcam, Cambridge, MA, USA) was used to assess intracellular ROS generation according to the manufacturer’s instructions. Raji cells were stained with 10 μM DCFH-DA for 30 min in the dark at 37°C. Subsequently, fluorescence signals were analyzed using a FACSCalibur flow cytometer (BD Biosciences).

### Mitochondrial membrane potential assay

Raji cells were incubated with JC-1 staining reagent (2 mL, Beijing Leagene Biotech. Co., Ltd., Beijing, China) for 20 min in the dark at 37°C. Cells were then washed three times with PBS, and JC-1 fluorescence was assessed using a FACSCalibur flow cytometer (BD Biosciences).

### Western blot assay

The BCA protein assay kit (Bio-Rad, Hercules, CA, USA) was used to determine protein concentrations. Equal amounts of total protein were separated by 10% SDS-PAGE and transferred onto a polyvinylidene difluoride (PVDF) membrane (Thermo Fisher Scientific). The membrane was then blocked in 5% skim milk at room temperature for 1 h and incubated with primary antibodies against Bax (cat. no. ab182733; 1:1000), Bcl-2 (cat. no. ab32124; 1:1000), cleaved caspase 3 (cat. no. ab32042; 1:1000), cleaved caspase 9 (cat. no. ab2324; 1:1000), p-Smad3 (cat. no. ab52903; 1:1000), Smad3 (cat. no. ab40854; 1:1000), E-cadherin (cat. no. ab227639; 1:1000), N-cadherin (cat. no. ab76011; 1:1000), Smad4 (cat. no. ab230815; 1:1000), Histone H3 (cat. no. ab1791; 1:1000), and anti-β-actin (cat. no. ab8227; 1:1000) at 4°C overnight. Next, membranes were incubated with horseradish peroxidase-conjugated secondary antibodies (cat. no. ab97051; 1:5000) at room temperature for 1 h. Immunoreactive bands were then visualized using an electrochemiluminescence instrument (Thermo Fisher Scientific). All antibodies were obtained from Abcam (Cambridge, MA, USA). β-actin and histone H3 were used as internal controls.

### Animal study

BALB/c nude mice (6–8 weeks old) were obtained from the Shanghai Slac Animal Center (Shanghai, China). Raji cells (5 × 10^6^ cells resuspended in 50 μL PBS) were injected subcutaneously into the left flanks of nude mice. When tumor volumes reached 180 mm^3^, animals were randomized into four groups: vehicle, 1 mg/kg HHT, 100 mg/kg curcumin, or HHT + curcumin. The vehicle group received normal saline only. HTT was administered in daily intraperitoneal (ip) injections at 1 mg/kg. Curcumin was administered via oral gavage at 100 mg/kg/day. Tumor volumes were calculated based on caliper measurements every week. After four weeks, animals were sacrificed under anesthesia according to the recommended procedures of the National Institutes of Health Guide for the Care and Use of Laboratory Animals. All animal experiments were approved by the First Affiliated Hospital of Zhejiang Chinese Medical University.

### TUNEL staining

Tumor tissues were fixed in 4% paraformaldehyde, embedded in paraffin, and cut into 5-μm sections. Cell apoptosis was assessed using a terminal deoxynucleotidyl transferase (TdT)-mediated dUTP nick end-labeling (TUNEL) assay with an *In Situ* Cell Apoptosis Detection Kit V (Boster Biological Technology Co. Ltd, Wuhan, China) according to the manufacturer’s protocol.

### Statistical analysis

All statistical analyses were performed using GraphPad Prism software (version 7.0, La Jolla, CA, USA). One-way analysis of variance (ANOVA) and Tukey’s tests were carried out for multiple group comparisons. All experiments were repeated in triplicate. Data are presented as the mean ± standard deviation (S.D.). ^*^P < 0.05 was considered statistically significant.

### Ethical statement

All animal experiments were performed with approval from the First Affiliated Hospital of Zhejiang Chinese Medical University.
