# Correction for: Curcumin in combination with homoharringtonine suppresses lymphoma cell growth by inhibiting the TGF-β/Smad3 signaling pathway

**DOI:** 10.18632/aging.203870

**Published:** 2022-01-30

**Authors:** Yu Zhang, Jingjing Xiang, Ni Zhu, Hangping Ge, Xianfu Sheng, Shu Deng, Junfa Chen, Lihong Yu, Yan Zhou, Jianping Shen

**Affiliations:** 1 Department of Hematology, The First Affiliated Hospital of Zhejiang Chinese Medical University, Hangzhou, Zhejiang 310006, PR China; 2 The First Medical College of Zhejiang Chinese Medical University, Hangzhou, Zhejiang 310053, PR China

Original article: Aging. 2021; 13:18757–18768. 18757-18768 . https://doi.org/10.18632/aging.203319

**This article has been corrected:** The authors have found a typing mistake in the FUNDING section: Zhejiang Provincial Natural Science Foundation (No.Y19H270018) should be (No.LY19H270004). The correct section is presented below.

## Funding 

Zhejiang Provincial Natural Science Foundation (No. LY19H270004, LY15H29004). Special project for the modernization of traditional Chinese medicine in Zhejiang Province (No. 2020ZX007). National TCM clinical research base construction project (No. 2015H0105). National Natural Science Foundation of China (No. 81800138).

